# Adenoids in School Children and Their Effect on the General System*Read at the Milam County Teachers’ Institute, October, 1911.

**Published:** 1912-01

**Authors:** G. B. Taylor

**Affiliations:** Cameron, Texas


For Texas Medical Journal.

### Adenoids in School Children and Their Effect on the General System.*

BY G. B. TAYLOR, M. D., CAMERON, TEXAS.

*History and Climatic Influence.*—In 1868 Meyer of Copenhagen first drew the attention of the medical profession to the clinical importance of adenoid growths.

Patients receive the greatest relief, both immediately and in the hereafter, by the removal of the growths. And incalculable numbers of individuals at this present moment are useful members of society, who would otherwise have become permanently deaf, besides suffering great inconvenience and discomfort. Those sufferers who have been neglected frequently bear ample evidence in their faces of the mischief done by adenoid growths in their childhood which persists throughout life. They are found in varying degrees of frequency in at least three parts of the world, namely, Europe, America and Asia. A warm climate seems to be less favorable to their development than a cold one. Along the equator where the temperature never varies between 75 to 80 degrees, and where it is dry and mountainous, only 5 out of 717 school children were found suffering, and not one out of 100 adults examined. On the contrary, in Greenland only 16 out of 60 Esquimaux children between the age of 4 and 12 were found to be free. These growths have probably existed from a very remote period of human history.

Several ancient Roman statues and busts show evidence of their existence as far as Roman art goes. The numerous likeness of the famous sculptor, Canova, are a proof of their existence at the beginning of the last century. They show the artist with open mouth, narrow nose and dull stolid look, and there is further testimony of his being deaf. They are met with at all ages from the new born up to seventy years of age.

Adenoid growths consist of a hypertrophy of the lymphoid tissue in the vault of the naso-pharynx, and is known as the pharyngeal tonsil and is especially well developed in children. It has been

*Read at the Milam County Teachers' Institute, October, 1911.

calculated that 90 per cent of children who have enlarged tonsils also have adenoid growths, but adenoid growths are present without enlarged tonsils. Statistics of school children indicate that from 20 to 30 per cent suffer from adenoids. The greater number of these display no symptoms sufficiently definite to attract general attention. Examination of 13,458 of Chicago's public school children showed that 5826, or about 42 per cent, had adenoids.

Energy, ambition, noble impulse and high ideals all succumb to pain and ill health and are frequently usurped by idleness and vice. Inability to apply themselves comfortably drives multitudes of children from school and thus reduces the intellectual and moral index of communal and national life. Therefore, a larger percentage than generally imagined possess adenoids, and they become a menace to hearing.

Cavanough of Chicago holds these growths responsible for 88 per cent of deafness. Tydings of Chicago states that at least 90 per cent of cases of adenoid vegetation there is involvement of the Eustachian tube with deafness in varying degree. If the deafness be marked and occur before the child has learned to talk, he will remain dumb, even should he become deaf at the age of 5 or 6 after he has acquired the faculty of speech, he will soon forget how to talk. There is no faculty upon which mental development is dependent to such extent as upon that of speech. Thought in its higher functions exists only through expression by means of language of which articulated speech is the most important manifestation. It is impossible for the child to reproduce what he has never heard, consequently two great stimulants are lacking, the power of understanding spoken language and the faculty of expression by means of speech. When to these handicaps are added the aprosexia and nervous instability usually in adenoid breathing subjects it is a small wonder that a diagnosis of idiocy or imbecility is often made.

When the deafness is complete after the removal of the primary cause, the treatment should be the same as that accorded any other deaf and dumb child. When there is even a slight vestige of hearing remaining it is often possible by persistent and careful effort to not only develop speech, but also to greatly improve the ability to hear. The condition occurring as the result of deafness due to adenoids may result either from slight deafness due to the middle ear disease, which prevents the child from hearing all sounds except those of a certain pitch or intensity, which he will, of course, reproduce as he hears them, or to faulty subjective audition, the objective hearing being normal. In the latter case he hears words correctly and believes that he reproduces them as heard. His subjective auditory center has become accustomed to receive faulty word impressions, and has lost or fails to develop its power of differentiation. This is dependent upon faulty kinesthetic derived from the peripheral musculature concerned in speech due to the presence of adenoids. For this reason after the removal of adenoids a special course of training directed toward the development of the kinesthetic and auditory word centers is necessary.

The aprosexia resulting from adenoids is probably responsible for more cases of backward and deviated mental development than any other cause. Unable to apply himself to his studies the child soon falls hopelessly behind his classmates and casted about for other means to keep his mind occupied, thereby getting himself into mischief of various kinds, and soon becomes a nuisance to his teacher and to his schoolmates. His school duties becoming hateful to him, he becomes truant with an enlarged field for his unrestrained activities, and finally coming into conflict with the authorities through his desire for constant change and excitement, ends in the juvenile court and the reformatory.

If he be of the phlegmatic type, content to plod without results, to stay where he is put, be becomes the dunce of his class. remaining in the same grade until either outwearing the overstrained patience of the teacher, or outgrowing his seat, he is advanced to the next grade, there to repeat the process. These are extreme cases, but they are to be found in every school and many others lesser only in degree in every class-room.

The hypoplastic child fundamentally unstable, and still further handicapped by adenoids, with their attendant train of evils, is prone to respond to an exaggerated degree to faulty environmental influences of all kinds, and requires the most careful study and attention to insure the advancement of which he is in a great number of instances capable. Many cases of pigeon breast and anterior transverse depressions are found in adenoid breathers. There is no doubt that children suffer in health owing to the muco-purulent discharge from the naso-pharynx entering the stomach, deranging the digestion and destroying the appetite.

Headaches and other nervous symptoms are not uncommon and are possibly due to pressure on the blood and lymphatic circulation in the naso-pharynx owing to the hyperaemia that usually exists in adenoids. Adenoids are undoubtedly a factor in the production of convulsions in young children owing to the imperfect oxygenation of the blood.

Dr. W. A. Fisher, ophthalmic surgeon to the eye, ear, nose and throat hospital of Chicago, has drawn attention to the following eye effects, which he firmly believes are secondary to adenoids:

1. Phlyctenular conjunctivitis, the most common.
2. So-called weak ulcer of the cornea, the non-inflammatory ulcer, which looks as if a small piece of the cornea surface has been gouged out.
3. *Eczmataus* of the cornea.
4. A peculiar irritability or sensitiveness of the retina, so that in the absence of corneal ulceration and all unusual causes of photophobia there is considerable difficulty in opening the eyes in a bright light.

These troubles are brought about by the general lowering of the general health produced by adenoids by the actual extension of the inflammatory process up the nasal duct to the eye in much the same way that adenoids produce deafness by extension of the inflammation up the Eustachian tube to the middle ear.

Adenoids occur in individuals who present symptoms of hypoplasia, resulting either from hereditary or development causes. Adenoids are factors in retardation by their mechanical action as source of reflex irritation and as impediment to proper development of the cerebral centers concerned in speech. Removal of adenoids is not sufficient in cases of marked defect to bring about restoration to normal. Special training and the institution of measures directed toward the amelioration of the underlying hypoplastic condition are necessary.

